# Editorial

**Published:** 1865-05

**Authors:** 


### Editorial.

Illinois State Medical Society.---The full record of proceedings of the recent meeting of our State Medical Society will be found in this number of the Examiner. The meeting was well attended. Though the standing committees all failed to report, yet the reports of special committees and volunteer papers were sufficient to occupy, pleasantly and profitably, all the time at the command of the meeting. The discussions were more than usually free, cordial, and interesting. The Committee of Arrangements discharged their duties with fidelity and good judgment. The visit to the State Normal University was very gratifying in every respect. The reception, on the part of the Principal and Faculty, was cordial and elegant; the institution, in all its departments, apparently under excellent management; and the location beautiful. We were glad to greet again among the members several who had been absent for the last three or four years, discharging the arduous and responsible duties of medical officers in the army. We hope our country may never *need* their services in the same capacity again.

Annual Meeting of the American Medical Association.—In the April number of the Examiner, we stated that whatever differences of opinion had existed in the minds of the profession in Boston, in relation to the approaching meeting of the Association, had been reconciled; and that the members would be as cordially received in Boston, on the 6th of June, as they had been in any other city in our country. As confirmatory of what we then said, we copy the following editorial from the *Boston Medical Surgical Journal*, of May 11th, 1865. We fully endorse the liberal sentiments it expresses, and hope they will be responded to by full delegations from every part of our great country:—

The American Medical Association.—The sixth of June, the day appointed for the meeting of the National Association, in Boston, is fast approaching, and all things now seem to combine to make the occasion one of unusual interest and complete success. The doubts which partly occupied the minds of some of us, as to the practicability of holding the meeting this year, while the storm of war still hung over the country, are dissipated in this new sunshine of peace, and our thoughts, no longer distracted by anxiety, can after four long years be again, and for the first time, wholly given to a consideration of our professional interests. Those who have so nobly represented us in the field, and who have not failed to sustain equally with our brave and victorious soldiers our national glory, are now returning, prepared to lay before us, at that time, the fresh results of their long labors, and it would be a lasting reproach to us did not the profession throughout the country make this the occasion of publicly expressing our appreciation of their services, and of giving them such a welcome back to our ranks as we know awaits the reception of the rest of our gallant army to its homes. To those, also, who were once so gladly met in these meetings, and who have as devotedly sacrificed themselves in the cause for which their people have so vainly fought, we extend, in the name of our national association, and our common interests in science, a cordial invitation.

The committees, to which have been entrusted the preparations for the reception and entertainment of our guests, have nearly matured their plans, and have met with the most ready coöperation from State and city authorities, and from all parties with whom they have communicated. The members of our profession will, without doubt, show the same willing spirit to assist the finance committee in its work when called upon, as we learn they will be during the coming week. From the entire unanimity in the profession, and the reputation which Boston possesses, as a centre of scientific and educational interests, there can be no doubt as to the nature of the reception or the attractive character of the entertainment which the city will give the Association. There is, indeed, but a single suggestion which we can offer to the committee, and this has, perhaps, been already anticipated; it is that invitations should be sent to the whole medical staff of the army, through the proper channels, to be present. It remains now for the various societies and institutions of the country, through their delegates, and for the chairmen of the scientific committees, to give to the coming meeting its true character, and we trust that all, far and near, will do their utmost to make it, as it promises to be, the most important one in the history of the Association.

---

ASSASSINATION OF PRESIDENT LINCOLN.---Of all the bloody and cruel tragedies of the last four years, none have startled the whole civilized world, and sent such a thrill of horror through every virtuous mind, as the deliberate murder of the late President of the United States. Surely the climax of wickedness has been reached. Let us hope the full cup of national affliction has been taken to its last drop. From this to the first day of June, the day set apart for national fasting and prayer, let every Christian in the land endeavor, by thorough self-examination to be prepared to make that day, not only one of fasting and prayer, but also the dawn of a full reunion of all Christian hearts, as well as of all the States of our great and, hitherto, highly favored country. As a matter of interest to all medical men, we append the following detailed account of the President's death and autopsy:---

The *Philadelphia Medical and Surgical Reporter*, of the 22d of April, contains a paper on the last hours of Abraham Lincoln, by C. S. Taft, Assistant-Surgeon, United States Army, which, as he says, "will doubtless prove of much interest to the profession, and may be relied on as correct in all particulars, the notes from which it was written having been submitted to comparison with others taken, and corrected, by the highest authority." Surgeon Taft was in the theatre when the President was shot; heard the pistol; "saw a wild-looking man jump from the box to the stage," and responded to the call for a surgeon. When he entered the box, the President was lying upon the floor, and "the respiration was inaudible and scarcely perceptible, and he was totally insensible." His "coat and vest had already been cut off, searching for the wound. The wound in the head was soon found, but, at that time, there was no oozing from it." When the President was removed to the house opposite, the wound was examined, "the finger being used as a probe, and the ball found to have passed beyond the reach of the finger, into the brain." The surgeon says:—

"I put a teaspoonful of diluted brandy between the lips, which was swallowed with much difficulty; a half teaspoonful administered ten minutes afterwards was retained in the throat, without any effort being made to swallow it. The respiration now became labored; pulse 44, feeble; eyes entirely closed, and left pupil much contracted, the right widely dilated; total insensibility to light in both."

The surgeon continues his report:—

"About 11:30 P.M., twitching of the facial muscles of the left side set in, and continued some fifteen or twenty minutes, and the mouth was drawn slightly to the same side. Sinapisms over the entire anterior surface of the body were ordered, together with artificial heat to the extremities.

"The wound began to ooze very soon after the patient was placed upon the bed, and continued to discharge blood and brain-tissue until 5:30 A.M., when it ceased entirely; the head, in the meantime, being supported in such a position as to facilitate the discharge. The only surgical aid that could be rendered, consisted in maintaining the head in such a position as to facilitate the discharge of the wound, and in keeping the orifice free from coagulum.

"Col. Crane, Surgeon, U.S.A., had charge of the head during a great part of the time, being relieved at intervals in this duty by myself. While the wound was discharging freely, the respiration was easy; but the moment the discharge was arrested from any cause, it became at once labored.

"It was also remarkable to observe the great difference in the character of the pulse, whenever the orifice of the wound was freed from coagulum, and discharged freely; thus relieving, in a measure, the compression. This fact will account for the fluctuations in the pulse, as given in the subjoined notes.

"At 2 A.M., an ordinary silver probe was introduced into the wound by the surgeon-general. It met an obstruction about three inches from the external orifice, which was decided to be the plug of bone driven in from the skull, and lodged in the track of the ball. The probe passed by this obstruction, but was too short to follow the track the whole length. A long Nélaton probe was then produced, and passed into the track of the wound for a distance of two inches beyond the plug of bone, when the ball was distinctly felt; passing beyond this, the fragments of the orbital plate of the left orbit were felt. The ball made no mark upon the porcelain tip, and was afterward found to be of exceedingly hard lead.

"Some difference of opinion existed as to the exact position of the ball, but the autopsy confirmed the correctness of the diagnosis upon first exploration. No further attempt was made to explore the wound.

"After the cessation of the bleeding from the wound, the respiration was stertorous up to the last breath, which was drawn at twenty-one minutes and fifty-five seconds past 7; the heart did not cease to beat until twenty-two minutes and ten seconds past 7. My hand was upon the heart, and my eye upon the watch of the surgeon-general, who was standing by my side, with his finger on the carotid.

"The decubitus during the whole time was dorsal, and the position on the bed diagonal; the length of the bedstead not admitting of any other position.

"The respiration during the last thirty minutes was characterized by occasional intermissions, no respiration being made for nearly a minute; but, by a convulsive effort, air would gain admission to the lungs, when regular, though stertorous, respiration would go on for some seconds, to be followed by another period of perfect repose.

"At these times, the death-like stillness and suspense were thrilling. The cabinet ministers and others surrounding the death-bed, watching, with suspended breath, the last feeble inspiration, and, as the unbroken quiet would seem to prove that life had fled, turn their eyes to their watches; then, as the struggling life within would force another fluttering respiration, heave deep sighs of relief, and fix their eyes once more upon the face of their dying chief.

"The wonderful vitality exhibited by the late President, was one of the most interesting and remarkable circumstances connected with the case. It was the opinion of the surgeons in charge that most patients would have died in two hours from the reception of such an injury, yet Mr. Lincoln lived from 10:30 P.M. until 7:22 A.M.

"The following observations of the pulse and respiration were noted down by Dr. A. F. A. King, at the bedside, and are correct. The pulse was counted by Acting-Assistant-Surgeon Ford:—

10:55—48.
11:06—45.
11:18—42, and weaker.
11:24—42, respirations 27 per minute, breathing quiet.
11:26— irregular, intermits occasionally.
11:30—45, respiration more frequently and vigorous.
11:32—45, stronger, respiration much more strong and stertorous.

11:37—48, respiration again silent and more feeble.
11:40—45.
11:43—45, respiration stertorous.
11:47—45, respiration 24, stertorous.
11:56—48, weaker.
12:10—48, irregularly intermittent.
12:18—48, same character.
12:27—54.
12:28—60.
12:29—66, intermittent.
12:38—66.
12:45—69, intermittent.
12:49—84, respiration 28.
12:56—66.
1:00—100.
1:15—92.
1:30—95.
2:10—60, respiration 34.
2:19—58.
2:32—54.
2:37—48.
2:54—48, much weaker, more thready; respiration feeble.
4:18—60, respiration 27, strong and stertorous.
5:40—64, thready, respiration 27.
6:10—60, hardly perceptible (Barnes), respiration 26, stertorous.
6:25—thready, not counted; respiration 22; inspirations jerking.
6:40—inspirations short and feeble; expirations prolonged and groaning; a deep, softly, sonorous, cooing sound at the end of each expiration, audible to bystanders.
6:45—respiration uneasy, choking and grunting; lower jaw relaxed; mouth open; a minute without a breath; face getting dark.
6:50—breathes again a little more at intervals; another long pause.
7:00---still breathing at long pauses.
7:20---died.

"About 1 P.M., spasmodic contractions of the muscles came on, causing proation of the fore-arms, the pectoral muscles seemed to be fixed, the breath was held during the spasm, and a sudden and forcible expiration immediately succeeded it.

"About the same time both pupils became widely dilated, and remained so until death.

"During the night, Drs. Hall, May, Liebermann, and nearly all the leading men of the profession in the city, tendered their services.

"AUTOPSY, FIVE HOURS AFTER DEATH.

"Present---Surgeon-General Barnes, Col. Crane, Dr. Stone, Assistant-Surgeon Woodward, U.S.A., Assistant-Surgeon Curtis, U.S.A., Assistant-Surgeon Notson, U.S.A., and Acting-Assistant-Surgeon Taft, U.S.A.

"The calvaria was removed, the brain exposed, and sliced down to the track of the ball, which was plainly indicated by a line of coagulated blood, extending from the external wound in the occipital bone, obliquely across from the left to the right through the brain to the anterior lobe of the cerebrum, immediately behind the right orbit. The surface of the right hemisphere was covered with coagulated blood. After removing the brain from the cranium, the ball dropped from its lodgment in the anterior lobe. A small piece of the ball, evidently cut off in its passage through the occipital bone, was previously taken out of the track of the ball, about four inches from the external wound. The hole made through the occipital bone was as cleanly cut as if done with a punch.

"The point of entrance was one inch to the left of the longitudinal sinus, and opening into the lateral sinus. The ball was flattened convex on both sides, and evidently molded by hand, in a Derringer pistol mold, as indicated by the rigid surface left by the nippers in clipping off the neck.

"The orbital plates of *both* orbits were the seats of comminuted fracture, the fragments being forced inward and the dura-mater covering them remaining uninjured. The double fracture was decided to have been caused by *contre coup*. The plug of bone driven in from the occipital bone was found in the track of the ball, about three inches from the external wound, proving the correctness of the opinion advanced by the surgeon-general and Dr. Stone, as to its nature, at the exploration of the wound before death.

"The ball and fragments, together with the fragments of the orbital plates, and plug from the occipital bone, were placed in the possession of Dr. Stone, the family physician, who marked and delivered them, pursuant to instructions, to the Secretary of State, who sealed them up with his private seal. The Nélaton probe used was also marked by me, and sealed up in like manner."

---

AMERICAN MEDICAL ASSOCIATION.—The American Medical Association will hold its Sixteenth Annual Meeting in the City of Boston, on Tuesday, June 6th, 1865.

The following Committees are expected to report:—

On Insanity—Dr. H. R. Storer, Mass., Chairman.

On Exsection and its connection with Conservative Surgery—Dr. Lewis A. Sayres, N. Y., Chairman.

On Drainage and Sewerage of Large Cities, and their Influence on Public Health—Dr. W. J. C. Duhamel, D. C., Chairman.

On Alcohol and its Relations to Man—Dr. G. E. Morgan, Maryland, Chairman.

On Quarantine—Dr. Wilson Jewell, Penn., Chairman.

On Medical Ethics—Dr. John A. Murphy, Ohio, Chairman.

On the Microscope—Dr. James M. Corse, Penn., Chairman.

On the Relations which Electricity sustains to the Causes of Disease—Dr. Squire Littell, Penn., Chairman.

On the Morbid and Therapeutic Effects of Mental and Moral Influences—Dr. A. B. Palmer, Mich., Chairman.

On the Causes of the Extinction of the Aboriginal Races of America—Dr. Geo. V. Suckley, Md., Chairman.

On the Causes and Treatment of Ununited Fractures—Dr. Frank H. Hamilton, N. Y., Chairman.

On Diphtheria—Dr. Lucius Clark, Ill., Chairman.

On the Uses and Abuses of Pessaries—Dr. James P. White, N. Y., Chairman.

On International Medical Ethics—Dr. J. Baxter Upham, Mass., Chairman.

On Climatology and Epidemic Diseases—Dr. C. W. Parsons, R. I., Chairman.

On Autopsies in Relation to Medical Jurisprudence—Dr. T. C. Finnell, N. Y., Chairman.

On so-called Spotted Fever—Dr. James J. Levick, Penn., Chairman.

On the Introduction of Disease by Commerce, and the Means of its Prevention—Dr. A. Nelson Bell, N. Y., Chairman.

On Patent Rights and Medical Men—Dr. David Prince, Ill., Chairman.

On Revision of Plan of Organization—Dr. N. S. Davis, Ill., Chairman.

On Specialists—Dr. J. Homberger, N. Y., Chairman.

On Compulsory Vaccination—Dr. A. N. Bell, N. Y., Chairman.

On Rank of Medical Corps in the Army—Dr. C. S. Tripler, U.S.A., Chairman.

On Rank of Medical Corps in the Navy—Dr. T. L. Smith, N. Y., Chairman.

WM. B. ATKINSON, *Permanent Secretary.*

### CITY MORTALITY.----STATISTICS FOR APRIL.

The mortality report for the month of April, has been prepared by Mr. M. P. Riordan, Assistant-Health-Officer, and will be found below. Notwithstanding the great influx of strangers into the city at this season of the year, the decrease in the number of deaths, when the past month is compared with the same month last year, is 48. The total number of deaths in April, 1864, was 298; while in April, 1865, it was only 250. Small-pox seems, also, to be on the decrease in the city. The number of deaths from this fell disease in April, 1864, was 35; in March, 1865, 16; while in April, 1865, only 4 died from small-pox. These are all reported from the West Division.

DISEASES.

| | | | |
|---|---|---|---|
| Asthma, | 1 | Congestive Fever, | 1 |
| Apoplexy, | 2 | Puerpural Fever, | 2 |
| Burned, | 1 | Spotted Fever, | 2 |
| Congestion of Brain, | 2 | Disease of the Heart, | 3 |
| Congestion of Lungs, | 1 | Inflammation of Brain, | 5 |
| Congestive Chills, | 1 | Inflammation of Bowels | 12 |
| Cholera Infantum, | 1 | Inflammation of Lungs, | 22 |
| Colds, | 4 | Inflammation of Kidneys, | 1 |
| Consumption, | 36 | Jaundice, | 2 |
| Croup, | 11 | Killed, | 3 |
| Cramps, | 19 | Liver Complaint, | 1 |
| Convulsions, | 13 | Measles, | 10 |
| Childbirth, | 3 | Marasmus, | 3 |
| Cancer of Stomach, | 3 | Old Age, | 7 |
| Drowned, | 2 | Paralysis, | 3 |
| Diabetes, | 1 | Puerpural Hemorrhage, | 1 |
| Diphtheria, | 4 | Pneumonia, | 1 |
| Dropsy, | 2 | Quinsy, | 1 |
| Diarrhœa, | 2 | Cerebro-Spinal Meningitis, | 3 |
| Erysipelas, | 2 | Suicide, | 2 |
| Found dead, | 2 | Small-Pox, | 4 |
| Bilious Fever, | 3 | Stillborn, | 10 |
| Lung Fever, | 2 | Teething, | 4 |
| Nervous Fever, | 2 | Unknown, | 7 |
| Scarlet Fever, | 8 | Whooping-Cough, | 3 |
| Typhoid Fever, | 8 | | |
| Water on Brain, | 1 | Total, | 250 |
| Typhus Fever, | 2 | | |

AGES OF THE DECEASED.—Under 5 years, 102; over 5 and under 10 years, 21; over 10 and under 20, 5; over 20 and under 30, 35; over 30 and under 40,

22; over 40 and under 50, 8; over 50 and under 60, 16; over 60 and under 70, 14; over 70 andunder 80, 7; over 80 and under 90, 2; stillborn, 10; unknown, 8. Total, 250.

NATIVITIES.

| | | | | | |
|---|---|---|---|---|---|
| Chicago, | 125 | Scotland, | 2 | Italy, | 1 |
| Canada, | 4 | Sweden, | 3 | Illinois, | 2 |
| Colored, | 5 | France, | 1 | Other States, | 42 |
| Belgium, | 1 | Germany, | 23 | Unknown, | 6 |
| England, | 5 | Holland, | 1 | Wales, | 1 |
| Ireland, | 25 | Norway, | 3 | | |
| Total, | | | | | 250 |

COMPARATIVE STATEMENT.

The following table shows the comparative number of deaths in the several divisions of the city for the month of April, 1864, and April, 1865:—

| April, 1864. | | April, 1865. | |
|---|---|---|---|
| South Division, | 117 | South Division, | 100 |
| West Division, | 108 | West Division, | 93 |
| North Division, | 70 | North Division, | 57 |
| Unknown, | 3 | | |
| Total, | 298 | Total, | 250 |

French Medical Students.—The number of students at the Paris School of Medicine for the present session, without including upwards of 100 foreigners, amounts to about 2000; being less by 142 than the number registered in 1863, when the new students entered in the books of the Faculty amounted to 357, whereas in the past year they were only 327; a small difference, it is true, but which shows, as in this country, a gradual and steady decrease in the numbers of the medical profession. The rejections average one in six.

Dr. Livingstone, the indefatigable medical explorer, is said to have devoted himself to a renewed series of African expeditions, and is planning a journey from the East Coast to the district lying between his most northern point on Lake Nyassa, and Burton and Speke's southernmost on Lake Tanganyka.

An Ounce of Quinine at a Dose.—Dr. Taussig, in a letter to a friend in London, relates a singular fact which occurred in Rome, where he resides, in December last. It is as follows:—

Dr. Hayler, a military medical man, visited in barracks a soldier, suffering from relapse of ague, and administered to him a small dose of sulphate of quinine. At the same time, he directed a man to fetch one ounce of the same remedy from the hospital, in order that he might have it in readiness for any emergency. The man received the bottle; but, supposing that it was ordered for the patient just mentioned, he took it to him.

In the presence of their comrades, they put the whole into a cup, adding sufficient water to make a paste of it; and the patient, although he found the medicine uncommonly bitter, did not leave off until he had swallowed it all.

Dr. Hayler, on learning that this enormous dose had been taken, at once visited the patient. The most careful investigation left no doubt of the fact; but, with all that, *incredibile dictu*, except a complete deafness and a kind of stupor, no other bad effect ensued, and no antidote was administered. He was directed to the hospital, where he remained for a week under observation, and left the establishment in the best state of health. The ague disappeared, probably never to return. I saw the man myself; he is a Swiss, named Albitz, aged 30, of small stature, and of a strong constitution.

It was not to be supposed that there was any important adulteration of the remedy in question, as all such preparations are subjected to a chemical investigation before they are admitted in the hospital dispensary.—*Medical Times.*

